# Thiopurine monotherapy is effective in ulcerative colitis but significantly less so in Crohn’s disease: long-term outcomes for 11 928 patients in the UK inflammatory bowel disease bioresource

**DOI:** 10.1136/gutjnl-2019-320185

**Published:** 2020-10-01

**Authors:** Evangelos Stournaras, Wendi Qian, Apostolos Pappas, You Yi Hong, Rasha Shawky, UK IBD BioResource Investigators, Tim Raine, Miles Parkes, UK IBD Bioresource Investigators, Tariq Ahmad

**Affiliations:** 1 Department of Gastroenterology, Cambridge University Hospitals NHS Foundation Trust, Cambridge, UK; 2 Cambridge Clinical Trials Unit, Cambridge University Hospitals NHS Foundation Trust, Cambridge, UK; 3 IBD BioResource, NIHR BioResource, Cambridge University Hospitals NHS Foundation Trust, Cambridge, UK; 4 Division of Gastroenterology and Hepatology, Department of Medicine, University of Cambridge, Cambridge, UK

**Keywords:** azathioprine, 6-mercaptopurine, crohn's disease, ulcerative colitis, tolerance

## Abstract

**Objective:**

Thiopurines are widely used as maintenance therapy in inflammatory bowel disease (IBD) but the evidence base for their use is sparse and their role increasingly questioned. Using the largest series reported to date, we assessed the long-term effectiveness of thiopurines in ulcerative colitis (UC) and Crohn’s disease (CD), including their impact on need for surgery.

**Design:**

Outcomes were assessed in 11 928 patients (4968 UC, 6960 CD) in the UK IBD BioResource initiated on thiopurine monotherapy with the intention of maintaining medically induced remission. Effectiveness was assessed retrospectively using patient-level data and a definition that required avoidance of escalation to biological therapy or surgery while on thiopurines. Analyses included overall effectiveness, time-to-event analysis for treatment escalation and comparison of surgery rates in patients tolerant or intolerant of thiopurines.

**Results:**

Using 68 132 patient-years of exposure, thiopurine monotherapy appeared effective for the duration of treatment in 2617/4968 (52.7%) patients with UC compared with 2378/6960 (34.2%) patients with CD (p<0.0001). This difference was corroborated in a multivariable analysis: after adjusting for variables including treatment era, thiopurine monotherapy was less effective in CD than UC (OR 0.47, 95% CI 0.43 to 0.51, p<0.0001). Thiopurine intolerance was associated with increased risk of surgery in UC (HR 2.44, p<0.0001); with a more modest impact on need for surgery in CD (HR=1.23, p=0.0015).

**Conclusion:**

Thiopurine monotherapy is an effective long-term treatment for UC but significantly less effective in CD.

**Significance of this study**

**What is already known on this subject?**

- Thiopurines are commonly used for maintenance of remission in steroid-dependent and steroid-refractory inflammatory bowel disease (IBD).
- Despite their widespread use, real-life data demonstrating durable effectiveness of thiopurines are sparse and are mostly derived from cohorts with modest numbers of patients and limited follow-up.
- In the era of cheaper biosimilar antitumour necrosis factor therapy, other biologics and newer small molecule therapies, the role of thiopurines in IBD management is increasingly being questioned.

**What are the new findings?**

- Using data from 11 928 patients with IBD treated with thiopurine monotherapy, we compared long-term effectiveness in ulcerative colitis (UC) and Crohn’s disease (CD) and their impact on need for surgery.
- Thiopurine monotherapy was effective for the duration of treatment in 52.7% of patients with UC but only 34.2% of patients with CD. On Kaplan-Meier analysis median duration on thiopurines in those in whom they were effective was 16 years for CD and 17 years for UC. Thiopurine monotherapy was more effective in UC than in CD in both prebiological and postbiological eras.
- Inability to tolerate thiopurines correlated with increased risk of surgery in UC (HR=2.44), with a more modest effect in CD (HR=1.23).

**Significance of this study**

**How might it impact on clinical practice in the foreseeable future?**

- In re-evaluating their current role in inflammatory bowel disease management, our study suggests that thiopurine monotherapy is effective in maintaining long-term clinical remission in ulcerative colitis. In Crohn’s disease, however, thiopurines are less effective as monotherapy particularly where there is perianal involvement. Here, there should be a lower threshold for biological therapy from outset±in combination with thiopurine.

## Background

The thiopurine analogues azathioprine (AZA) and 6-mercaptopurine (6MP) have held a long-standing place in the management of inflammatory bowel disease (IBD). In both ulcerative colitis (UC) and Crohn’s disease (CD) they are used for maintaining remission in steroid-dependent or steroid-refractory disease.^1–3^ However, in recent years increasing emphasis has been placed on the known toxicity profile of thiopurines and, with increasing numbers of biological therapies including cheaper biosimilars available, some authorities are questioning their role.

Randomised controlled trials (RCTs) of thiopurines in IBD from the 1970s to the 90s suggested efficacy in both UC and CD but, as was typical for RCTs of this era, the number of patients included was small and follow-up limited.^4–10^ More recently, the AZathioprine for Treatment or Early Crohn's disease in adults (AZTEC) and Résultat de l'Adjonction Précoce d'ImmunoDépresseurs (RAPID) strategy trials assessed the efficacy of azathioprine in maintaining remission when used very soon after diagnosis in CD. Neither demonstrated benefit of very early vs conventional use of thiopurines.^11 12^

To gain further insight, single-centre retrospective studies in population-based cohorts have investigated the real-world outcomes of AZA and 6MP treatment in IBD and suggested 40%–60% effectiveness.^13–18^ Again, however, the numbers involved have been relatively modest and/or the follow-up relatively short, and few studies have compared effectiveness in UC versus CD.^13 15–18^

The effect of thiopurines in reducing surgery has also been studied. Early need for thiopurine clearly defines patients at increased risk for colectomy in UC,^19^ and the inability to tolerate thiopurine has also been associated with increased colectomy risk.^20 21^ In CD results have been more mixed. One French study suggested that thiopurines reduced need for surgery while another did not.^22 23^ In two UK studies reduced need for surgery correlated with increased thiopurine use—with a national cohort study showing 44% less surgery in patients on thiopurine for at least 6 months.^24 25^

There is little consensus regarding how long thiopurine treatment should be continued. One retrospective series suggested 4 years,^26^ but relapse following thiopurine cessation is common and response cannot always be recaptured.^13^

With more biologic and newer small molecule therapies now available some question the role of thiopurine monotherapy in IBD, asserting that the newer agents are more effective and safer. Biological therapies are, however, not without their own problems including non-response and particularly loss of response over time^27–30^; and the Janus-kinase inhibitor tofacitinib has also been associated with safety signals.^31 32^ Additionally, costs of biologic and JAK inhibitor therapies remain a bar in many healthcare settings.

Here, we present the outcomes of thiopurine use in the UK IBD BioResource—launched in 2016 as part of the UK National Institute for Health Research BioResource and encompassing a large cohort of ‘recallable’ patients on whom clinical details were ascertained at enrolment.^33^ We designed the study to investigate the long-term effectiveness of thiopurine monotherapy in UC and CD and explore the impact of thiopurine tolerance on the need for surgery.

## Methods

We undertook a retrospective analysis of the outcomes of thiopurine treatment (AZA or 6MP) for patients with CD or UC in the IBD BioResource. Those with IBD-unclassified (IBDU) were included with UC. Patients were involved in the study design. All participants provided signed consent. The IBD BioResource is currently recruiting in 104 hospitals UK-wide. At a data lock taken on 14 January 2020, 31 481 patients had been enrolled.

Structured IBD phenotype data, including drug therapy outcomes and surgeries, were ascertained at IBD BioResource enrolment by research nurses and clinicians using a combination of case note review, patient interview and patient questionnaire. Periodic data validation exercises are undertaken with independent reassessment of phenotype data. Most sites recruited unselected consecutive patients with IBD attending clinic, the overall numbers recruited at each site varying according to duration of study setup and resources available.

For each subject in the current study, the outcome of historic or current AZA or 6MP treatment was assessed by the question ‘Was the treatment effective?’, with responses being empirically classified into one of the following seven categories:

1. Yes.
2. No (on therapeutic dose >4 months—did not work).
3. Unable to assess (on therapeutic dose <4 months).
4. Unable to assess (unable to tolerate).
5. Worked for <12 months then lost response.
6. Worked for >12 months then lost response.
7. Response not known (eg, started antitumour necrosis factor (TNF) at same time, partial response only etc).

Our primary aim was to identify the proportion of patients treated with thiopurine monotherapy in whom this was deemed effective, satisfying both (A) response of ‘yes’ (option 1) to the question ‘Was the treatment effective?’ and (B) patient had not needed escalation to biologic therapy or IBD surgery for the duration of thiopurine therapy. Patients were excluded if the treatment response data were missing; and those started on anti TNF therapy at thiopurine initiation or undergoing surgery in the year of thiopurine initiation (where the drug may have been used as postsurgical prophylaxis) were excluded since we could not assess effectiveness of thiopurine monotherapy in these groups. UC patients who had undergone colectomy prior to thiopurine initiation were also excluded; as were cases where missing dates did not allow us to confirm whether biological therapy/surgery overlapped the indicated period of thiopurine monotherapy.

The effectiveness of thiopurine monotherapy was also investigated in terms of time from initiating thiopurine to requirement for treatment escalation with biological therapy or surgery, whichever occurred first.

To investigate whether thiopurine therapy has an impact on need for surgery in UC and CD, the time from treatment initiation to surgery was compared between patients able to tolerate thiopurine versus those unable to tolerate thiopurine. Only patients who were deemed unable to tolerate any thiopurine at any dose were labelled as ‘intolerant’ in this analysis (eg, this group did not include people unable to tolerate AZA but able to tolerate 6MP; or those able to tolerate thiopurine following dose reduction±concomitant allopurinol).

Descriptive statistics of the clinical characteristics were calculated, with continuous variables presented as medians (25th–75th percentile); and categorical variables as frequencies and percentages. Duration of thiopurine monotherapy was calculated from start to end of treatment or censored for participants who were still on thiopurine monotherapy at the time of data lock, and summarised using Kaplan-Meier estimates. Adverse event incidence was compared between UC and CD by X^2^ testing. The Cochran-Mantel-Haenszel (CMH) test was applied to compare the effectiveness of thiopurine monotherapy between UC and CD, using stratified treatment initiation time periods grouped 5 yearly. Comparison of the effectiveness of thiopurine between UC and CD was also assessed using a multivariable logistic regression with covariates of age at diagnosis, gender, smoking history, treatment era and time from diagnosis to thiopurine initiation. Kaplan-Meier methods were applied for the time to treatment escalation with biological therapy or surgery. The differences of distributions between UC and CD were compared using the log-rank test. Logistical regression models were applied to explore the correlation between the thiopurine monotherapy effectiveness and the clinical characteristics within UC and CD using the same covariates as above but also including disease location. Similar analyses were performed using the Cox regression models for the time to surgery outcome. Due to the large size of the dataset, a conservative prespecified p value threshold of less than 0.005 was considered statistically significant. Statistical analyses were performed using the SAS V.9.4.

## Results

### Patient population

**Figure 1:**
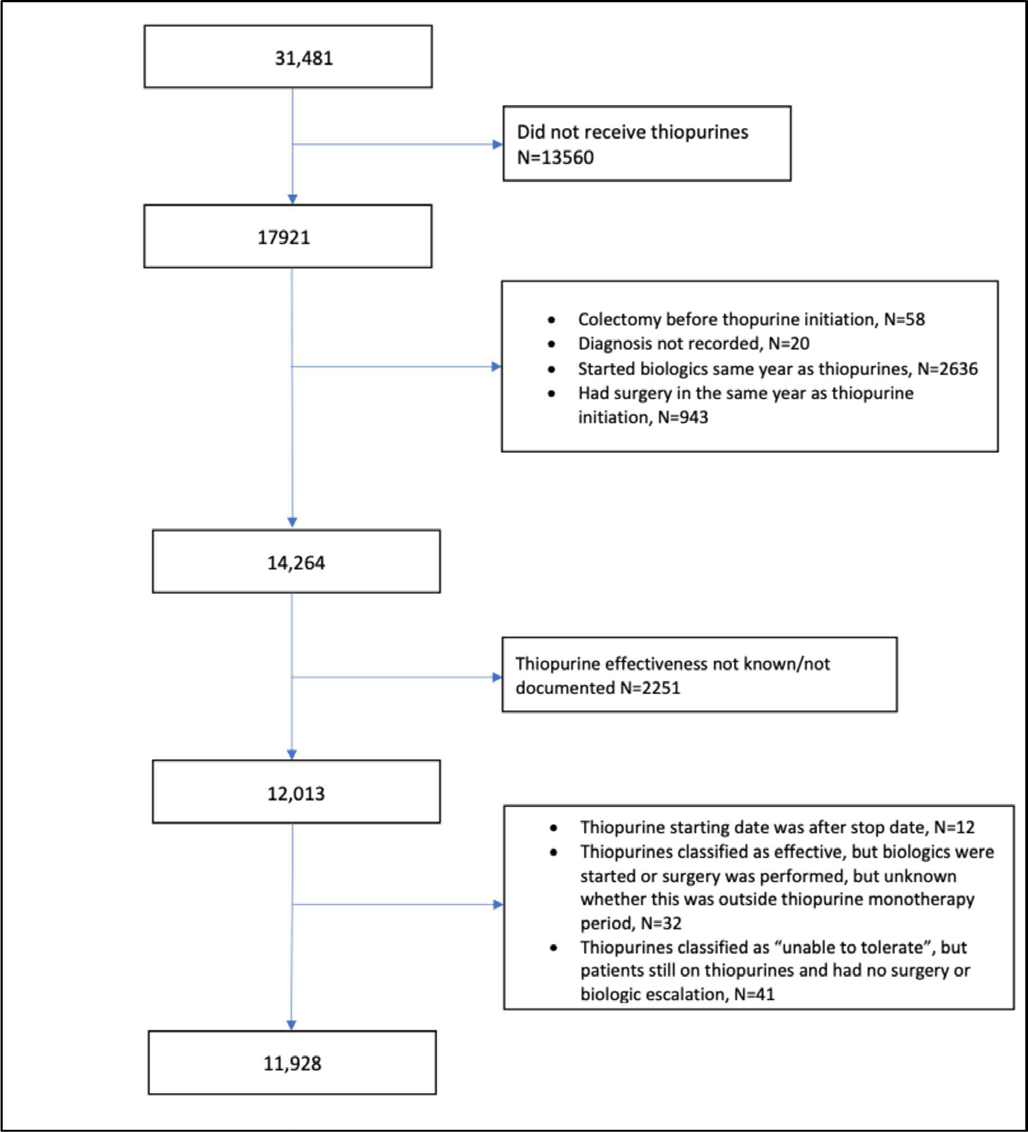
Study flow chart.

Of the 31 481 participants in the IBD BioResource at the data lock, 17 921 (56.9%) had been treated with a thiopurine for IBD (71.9% with CD; 43.8% with UC), either as monotherapy or combined with anti-TNF therapy. A total of 11 928 met the criteria for assessment of the effectiveness of thiopurine monotherapy (6960 CD and 4968 UC (including 251 IBDU)). Hereafter all analyses refer to this cohort of 11 928 subjects (figure 1).

Patient characteristics are presented in table 1. Median age at CD/UC diagnosis was 26/33 years. Median time from diagnosis to data capture was 12 years (total follow-up 99 555 patient-years/total thiopurine exposure 40 807 patient-years) for CD and 10 years (total follow-up 60 413 patient-years/thiopurine exposure 27 325 patient-years) for UC.

**Table 1:** Demographic and clinical characteristics and adverse reactions in the 11 928 patients in the IBD BioResource treated with thiopurine monotherapy

	CD		UC	
**Age at diagnosis**	26(19–39)		33 (24–45)	
Gender				
Male	3181 (45.8%)		2615 (52.7%)	
Female	3768 (54.2%)		2347 (47.3%)	
Smoking status at diagnosis				
Never smoked	3387 (51.9%)		2538 (54.5%)	
Smoking at diagnosis	2236 (34.2%)		673 (14.5%)	
Ex-smoker	909 (13.9%)		1443 (31.0%)	
**Disease location**				
CD				
Ileal	2487 (37.0%)			
Colonic	1747 (26.0%)			
Ileo-colonic	2413 (35.9%)			
Exclusive upper GI Crohn’s	75 (1.1%)			
Perianal involvement	1989 (30.2%)			
UC				
Proctitis (E1)			379 (8.5%)	
Left sided (E2)			2143 (48.3%)	
Extensive (E3)			1917 (43.2%)	
**Tolerated thiopurine**	5382 (77.3%)		4064 (81.8%)	
**Adverse reactions** (X^2^ test between groups unable to tolerate thiopurine)	**Whole cohort**	**Intolerant group** (had to discontinue thiopurines)	**Whole cohort**	**Intolerant group** (had to discontinue thiopurines)
	N (%)	N (%)	N (%)	N (%)
Abdominal pain (p=0.04)	216 (3.2)	134 (2.0)	115 (2.3)	71 (1.5)
Deranged LFT (**p=0.0003**)	326 (4.8)	146 (2.1)	369 (7.5)	158 (3.2)
Flu-like symptoms (p=0.01)	177 (2.6)	99 (1.5)	119 (2.4)	70 (1.4)
Leucopenia (p=0.45)	145 (2.1)	38 (0.6)	123 (2.5)	32 (1.4)
Nausea/vomiting (p=1.32)	793 (11.6)	434 (6.4)	563 (11.5)	286 (5.8)
Pancreatitis (**p<0.0001**)	235 (3.4)	182 (2.7)	78 (1.6)	57 (1.2)
Other (p=0.53)	1490 (21.4)	712 (10.2)	1056 (21.6)	488 (10)

CD, Crohn’s disease; IBD, inflammatory bowel disease; LFT, liver function tests; UC, ulcerative colitis.

### Thiopurine treatment and tolerability

Of the 11 928 subjects treated with thiopurine 11 239 (94.2%) received AZA and 2698 (22.6%) 6MP; this included 2009 (16.8%) who received both at different time points. A total of 2984 (26.6%) subjects treated with AZA were unable to tolerate it and had to stop. Of those exposed only to 6MP, 138/689 (20.0%) experienced an adverse effect and consequently stopped. Of 11 928, 2482 (20.8%) could not tolerate either thiopurine at any dose. A total of 1327 patients were exposed to both AZA and 6MP after intolerance of their first thiopurine, of whom 640/1327 (48.2%) were able to tolerate the second thiopurine.

**Figure 2:**
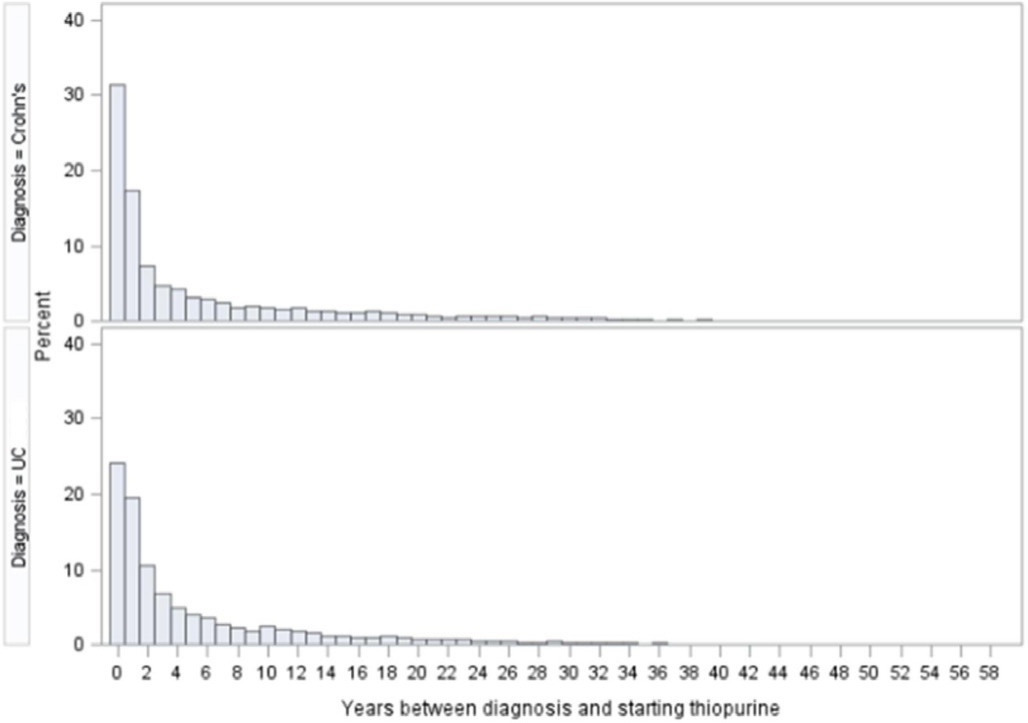
Time (years) from IBD diagnosis to thiopurine initiation in the 11 928 subjects. IBD, inflammatory bowel disease.

Median overall duration of thiopurine treatment was 8 (1–25) years for CD and 9 (1–24) years for UC. Median time from diagnosis to thiopurine initiation was 2 years for both CD and UC, with 46.7% starting thiopurine within 2 years of diagnosis (figure 2). A total of 2905 (41.7%) of those with CD and 1377 (27.7%) with UC received biological therapies after attempted treatment with thiopurine monotherapy.

Adverse events were consistent with those reported previously (table 1). Nausea/vomiting and deranged liver function tests were the most common. Permanent thiopurine discontinuation due to pancreatitis was more common in CD than UC (2.7% vs 1.2%, X^2^ p<0.0001), whereas treatment-limiting hepatotoxicity was more common in UC (3.2% vs 2.1% in CD, p=0.0003).

### Effectiveness of thiopurine monotherapy

Of 11 928 subjects with IBD treated with thiopurine monotherapy, it was reported to have been effective, without the need for escalation to biologic therapy or need for surgery for the duration of thiopurine treatment, in 4995 (41.9%). It was effective for UC in 2617/4968 (52.7%) of patients; and a lower proportion of patients with CD: 2378/6960 (34.2%).

Using the CMH test to stratify by treatment initiation era, thiopurine monotherapy appeared more effective in UC than CD for all 5-year chronological periods (RR 1.40, 95% CI 1.35 to 1.45, p<0.0001) (online supplementary figure 1). The absolute effectiveness of thiopurine monotherapy appeared to decrease in UC and increase in CD in more recent cohorts (online supplementary figures 2 and 3). We incorporated treatment era along with other possible confounding factors in a multivariable logistic regression analysis. This again demonstrated that thiopurine monotherapy was less effective in CD than UC (OR 0.47, 95% CI 0.43 to 0.51, p<0.0001), after adjusting for era of starting thiopurines, time from diagnosis to thiopurine initiation, age at diagnosis and smoking history (table 2).

10.1136/gutjnl-2019-320185.supp1 Supplementary data

10.1136/gutjnl-2019-320185.supp2 Supplementary data

10.1136/gutjnl-2019-320185.supp3 Supplementary data

**Table 2:** Multivariable analysis of factors affecting thiopurine monotherapy effectiveness

	OR	95% CI	P value
Diagnosis			
CD	**0.47**	0.43 to 0.51	**<0.0001**
UC	1	Referent	
Treatment period	**1.06**	1.02 to 1.09	**0.0008**
Time from diagnosis to thiopurine initiation	**1.02**	1.01 to 1.02	**<0.0001**
Age at diagnosis	**1.01**	1.01 to 1.02	**<0.0001**
Gender			
Female	1.06	0.97 to 1.14	0.19
Male	1	Referent	
Smoking history			
No	1.12	1.03 to 1.22	0.007
Yes	1	Referent	

Significant p values shown in bold.

CD, Crohn’s disease; UC, ulcerative colitis.

### Clinical characteristics and response to thiopurine monotherapy

For CD, in a multivariable analysis after controlling for confounding factors, thiopurine therapy appeared more effective in patients with colonic as opposed to ileocolonic involvement (OR 1.60, 95% CI 1.38 to 1.86, p=0.002) and older patients (1.01 per year, 95% CI 1.01 to 1.02, p<0.0001). By contrast, treatment initiation ≤1 year after IBD diagnosis (OR 0.69, 95% CI 0.60 to 0.78, p<0.0001) and perianal disease (OR 0.70, 95% CI 0.61 to 0.80, p<0.0001) were associated with lower thiopurine effectiveness (table 3). No correlation was found with gender or smoking history.

**Table 3:** Regression analysis of factors influencing thiopurine effectiveness in CD and UC patients

	CD				UC			
	Univariable		Multivariable		Univariable		Multivariable	
	OR (95% CI)	P value	OR (95% CI)	P value	OR (95% CI)	P value	OR	P value
Age at diagnosis	**1.02 (1.01 to 1.02**)	**<0.0001**	**1.01 (1.01 to 1.02**)	**<0.0001**	1 (1.00 **to** 1.01)	0.49		
Gender		0.08				0.72		
Male	1.09 (0.99 **to** 1.20)				1.02 (0.91 **to** 1.14)			
Female	Referent				Referent			
Smoking history		0.34				0.12		
No	0.95 (0.86 **to** 1.05)				1.01 (0.98 **to** 1.23)			
Yes	Referent				Referent			
Treatment era			**1.2 (1.14 to 1.25**)	**<0.0001**			**0.88 (0.83 to 0.93**)	**<0.0001**
Time from diagnosis to thiopurine initiation								
<1 year	**0.72 (0.64 to 0.82**)	**<0.0001**	**0.69 (0.60 to 0.78**)	**<0.0001**	0.87 (0.76 **to** 1.0)	0.36		
≥1 and <2 years	0.92 (0.80 **to** 1.06)	0.24	0.81 (0.70 **to** 0.95)	0.81	0.86 (0.74 **to** 1.0)	0.27		
≥2 years	Referent							
Disease location (CD)								
Ileal	1.28 (1.14 **to** 1.45)	0.57						
Colonic	**1.61 (1.42 to 1.84**)	**0.008**	**1.6 (1.38 to 1.86**)	**0.002**				
Exclusive upper GI	1.53 (0.96 **to** 2.46)	0.44						
Ileo-colonic	Referent							
Perianal involvement								
Yes	**0.67 (0.59 to 0.75**)	**<0.0001**	**0.7 (0.61 to 0.80**)	**<0.0001**				
No	Referent							
Disease location (UC)								
Proctitis (E1)					**1.47 (1.17 to 1.84**)	**0.0006**	**1.56 (1.23 to 1.98**)	**0.0002**
Left sided (E2)					1.01 (0.89 **to** 1.14)	0.01		
Extensive (E3)					Referent			

Significant p values shown in bold.

CD, Crohn’s disease; UC, ulcerative colitis.

For UC, proctitis was associated with higher effectiveness (OR=1.56 vs extensive disease, 95% CI 1.23 to 1.98, p=0.0002) (table 3).

### Duration of effect

For participants in whom thiopurine monotherapy was deemed effective, over 70% were still on this treatment at the time of our data lock. Median treatment duration on Kaplan-Meier analysis of this group was 16 (95% CI 15 to 19) years for CD and 17 (95% CI 15 to 20) years for UC (online supplementary figure 4).

10.1136/gutjnl-2019-320185.supp4 Supplementary data

**Figure 3:**
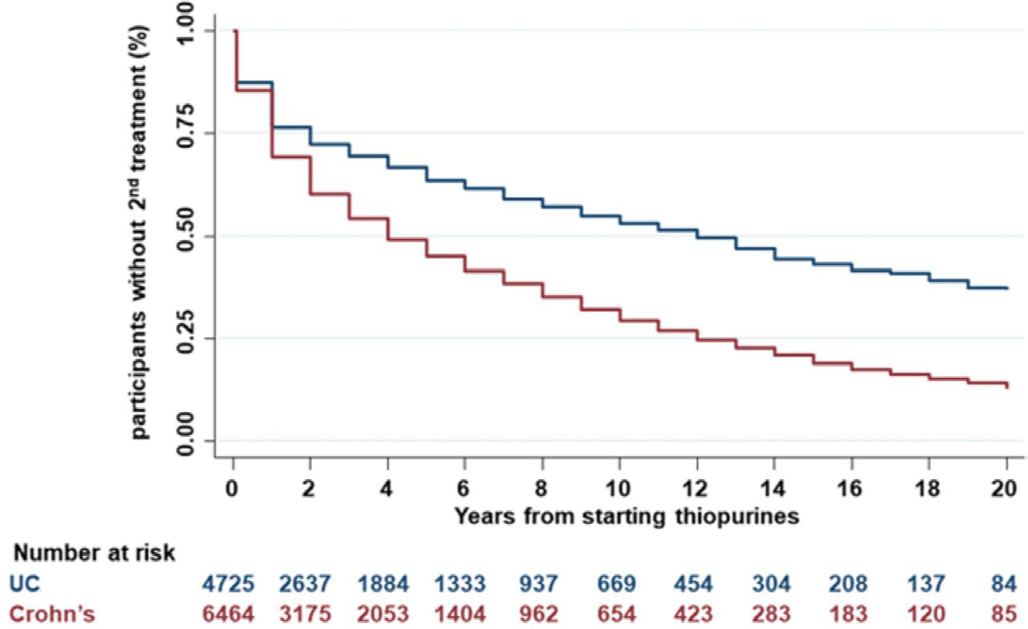
Kaplan-Meier plots of time during which thiopurine monotherapy was effective in UC and CD, without requirement for treatment escalation (p<0.0001 for log-rank test). CD, Crohn’s disease; UC, ulcerative colitis.

We performed a time-to-event analysis for patients on thiopurine monotherapy assessing time to escalation to biological therapy or surgery. A total of 11 189 (6464 CD, 4725 UC) participants were included with 739 excluded due to missing data. Of patients continuing thiopurine 4907 (44%) required treatment escalation after a median time of 4 (95% CI 4 to 5) years in CD and 12 (95% CI 11 to 13) years in UC (p<0.0001 for log-rank test) (figure 3). This remained significant after adjusting for treatment period and clinical characteristics using the Cox regression model (p<0.0001). Of patients initiated on thiopurine monotherapy, at 1 and 3 years 69% and 54% of those with CD, and 76% and 69% with UC, respectively, remained on thiopurine and had not required treatment escalation to biological therapy or surgery (online supplementary table 1).

10.1136/gutjnl-2019-320185.supp5 Supplementary data

### Effect of thiopurine therapy on surgery in UC

**Figure 4:**
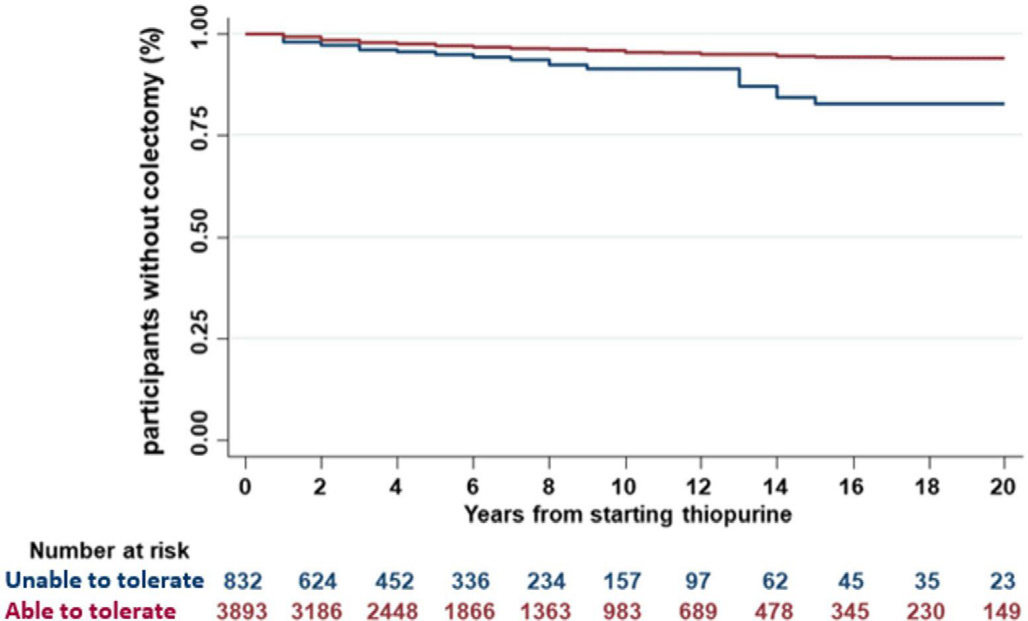
Kaplan-Meier plot showing the proportion of patients with UC not requiring colectomy relative to time since initiation of thiopurines, stratified by ability to tolerate thiopurines. Patients unable to tolerate thiopurine had a significantly shorter time to colectomy (log-rank <0.0001). UC, ulcerative colitis.

Of the 4968 patients with UC in whom thiopurine monotherapy was assessed, 219 (4.4%) had required colectomy—92 for acute severe colitis, 92 for medically refractory disease, 12 for cancer/dysplasia and 23 with reasons not documented. We analysed the proportion of patients requiring colectomy according to tolerance of thiopurine treatment: 63/904 (7.0%) of patients intolerant of thiopurine required colectomy compared with 156/4064 (3.8%) of those able to tolerate thiopurines. Time to colectomy was shorter in individuals unable to tolerate vs able to tolerate thiopurine (figure 4, log-rank <0.0001). In a Cox proportional hazards model, the HR for colectomy was 2.44, 95% CI 1.71 to 3.50 (p<0.0001) in individuals unable to tolerate thiopurine. Extensive colitis and younger age at diagnosis also correlated with increased risk of colectomy (table 4).

**Table 4:** Cox regression analysis of factors affecting time to colectomy in UC patients

	P value	HR	95% CI
Able to tolerate thiopurines			
No	**<0.0001**	**2.44**	1.71 to 3.50
Yes		1	Referent
Age at diagnosis	**<0.0001**	**0.99**	0.96 to 0.99
Treatment period	0.46	1.06	0.91 to 1.23
Time from diagnosis to thiopurine initiation	**0.002**	**0.96**	0.93 to 0.98
Gender			
Female	0.28	0.84	0.60 to 1.16
Male		1	Referent
Smoking history			
No	0.48	0.88	0.62 to 1.25
Yes		1	Referent
Disease location			
Proctitis (E1)	0.54	0.75	0.30 to 1.90
Left sided (E2)	**Referent**	**1**	Referent
Extensive (E3)	**<0.0001**	**2.58**	1.78 to 3.72

UC, ulcerative colitis.

Treatment with biological therapies also differed between groups. Of patients with UC who were unable to tolerate thiopurines 365/904 (40.4%) subsequently received biological therapy, compared with 1012/4064 (24.9%) of those able to tolerate thiopurines (p<0.0001). Thus, patients with UC unable to tolerate thiopurines required earlier colectomy despite increased use of biological therapies.

### Effect of thiopurine therapy on surgery in CD

Unlike with UC, patients with CD with a surgical procedure prior to thiopurine initiation were included in this analysis, because reoperation is common in the natural history of CD. In total, 3077/6960 patients (44.2%) treated with thiopurine monotherapy underwent surgery for CD. 1517/6960 (21.8%) had an operation after thiopurine initiation.

**Figure 5:**
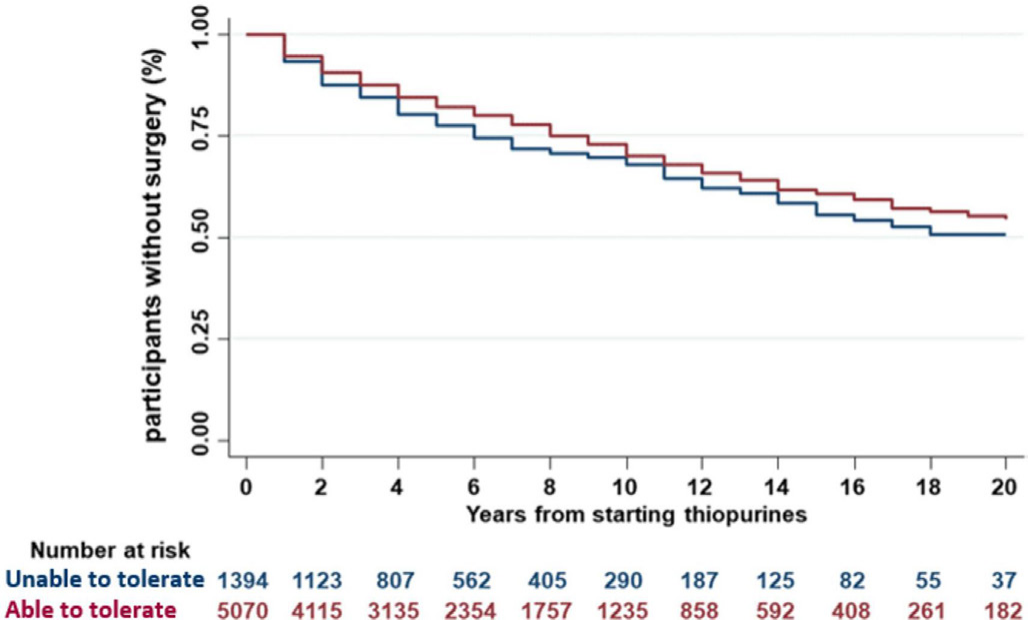
Kaplan-Meier plot showing the proportion of patients with CD not requiring surgery relative to time since initiation of thiopurines, stratified by ability to tolerate thiopurines. Patients unable to tolerate thiopurine had a significantly shorter time to surgery (log-rank=0.002). CD, Crohn’s disease.

A total of 1578 patients with CD could not tolerate thiopurine treatment. Their time to surgery was modestly shorter compared with the 5382 patients with CD able to tolerate thiopurines in the Kaplan-Meier analysis (log-rank=0.002 - figure 5). Inability to tolerate thiopurine was associated with risk of surgery in a Cox proportional hazards model (HR 1.23, 95% CI 1.08 to 1.40, p=0.0015). The latter also identified reduced risk of surgery in patients with CD where only the colon was involved (table 5), as previously reported.

**Table 5:** Cox regression analysis of factors affecting time to surgery in CD patients

	P value	HR	95% CI
Able to tolerate thiopurines			
No	**0.0015**	**1.23**	1.08 to 1.40
Yes		1	Referent
Treatment period	0.92	1	0.96 to 1.05
Time from diagnosis to thiopurine initiation	**0.0001**	**0.99**	0.98 to 0.99
Age at diagnosis	**0.0001**	**0.99**	0.986 to 0.995
Gender			
Female	0.05	1.11	1.00 to 1.24
Male		1	Referent
Smoking history			
No	0.06	0.89	0.80 to 1.01
Yes		1	Referent
Disease location			
Ileal	0.058	1.13	0.99 to 1.27
Colonic	**<0.0001**	**0.5**	0.43 to 0.58
Exclusive upper GI	0.46	1.22	0.71 to 2.07
Ileocolonic		1	Referent
Perianal involvement			
No	**<0.0001**	**0.52**	0.47 to 0.59
Yes		1	Referent

Significant p values shown in bold.

CD, Crohn’s disease.

Use of biological therapy again differed between the two groups. 782/1578 (49.6%) patients with CD unable to tolerate thiopurines were escalated to biological therapy, compared with 2123/5382 (39.4%) of patients able to tolerate these drugs (p<0.0001).

## Discussion

Thiopurines are commonly used in the treatment of IBD, but their role is increasingly questioned given the ever-expanding range of biologic therapy options, the introduction of modestly priced biosimilar anti-TNF, increasing concerns about the tolerability and safety of thiopurines and a lack of data regarding efficacy.^11 12^

We report the first large-scale study in the biological era assessing clinical practice and patient-level outcomes of IBD treatment with thiopurines. Long-term outcome data remain sparse for all IBD treatments, but durability is key in therapy planning for CD and UC given their chronic nature. The most striking finding from our study was the evidence that thiopurines provided an effective long-term treatment for 52.7% of patients with UC, without need for escalating to biological therapy or surgery; but that durable effectiveness in CD was lower at 34.2%. These results include all individuals in whom AZA and 6MP were initiated and do not exclude those intolerant of thiopurines.

Our finding of increased effectiveness for thiopurine monotherapy in UC compared with CD was consistent when we performed a multivariable logistic regression correcting for baseline differences (CD vs UC, OR 0.47, 95% CI 0.43 to 0.51). The same conclusion was obtained when any missing covariates were imputed (estimated OR 0.50, 95% CI 0.46 to 0.54). Perianal involvement is an established risk factor for thiopurine treatment failure, corroborated by the results of our study (OR 0.70, 95% CI 0.61 to 0.80). We undertook a sensitivity analysis testing whether the lower effectiveness in CD overall might be due to poor response in the perianal group. Even after excluding patients with perianal CD, thiopurine monotherapy remained less effective in patients with purely luminal CD as compared with those with UC (OR 0.52, 95% CI 0.47 to 0.56, p<0.0001). In a further analysis using a different definition of effectiveness, which considered time to treatment escalation or surgery, we again found evidence of increased thiopurine effectiveness in UC compared with CD (figure 3). Finally, the protective effect against surgery in those tolerant of thiopurines compared with those intolerant was again more apparent in UC than CD (figures 4 and 5).

Since treatment landscapes and expectations have changed over time, we wanted to understand whether treatment era might have influenced reported outcomes. While treatment era was a significant covariate in our multivariable model, thiopurines appeared more effective in UC than CD in all time periods (online supplementary figure 1), including during the periods after the arrival of licensed and reimbursed biologics. The apparent trend to reduced comparative effectiveness of thiopurine monotherapy in UC versus CD over time was mainly driven by seeming increased effectiveness in CD (online supplementary figure 3). Several factors might contribute to this, some of which reflect the potential biases inherent in data derived retrospectively for a cohort study such as ours. In CD, which often progresses to needing surgery over time (eg, for development of strictures), duration of follow-up for patients recently started on thiopurine may have been insufficient for the need for surgery to accrue. Requirement for surgery indicated ‘ineffective’ treatment in the definition we used in our primary effectiveness analysis—hence potentially inflating apparent effectiveness in recently started cohorts compared with cohorts on therapy for longer. In UC the opposite selection bias may have impacted, with under-representation in medical clinics (where most IBD BioResource participants are recruited) of patients that had undergone colectomy years before, leading to inflation of apparent effectiveness rates of thiopurine among those still attending. Finally, in the postbiological era patients and physicians may have altered expectations and be more ready to escalate therapy for side effects or delayed treatment action of thiopurines. In this context, although changes in UK reimbursement rules greatly improved access to biologics for UC after 2015, any changes in apparent effectiveness of thiopurine monotherapy after this date were modest (online supplementary figure 2). In summary, although we recognise the inherent limitations in our retrospective data series, we do not believe that any of the potential biases we have identified would explain our key finding of increased effectiveness in UC compared with CD.

Our multivariable analysis also identified specific patient subgroups most likely to respond to treatment with thiopurine monotherapy. These included older patients with more distal UC, or colonic (but not perianal) CD. For both UC and CD patients starting treatment longer after diagnosis responded better. This may reflect disease severity bias as patients with aggressive disease often escalate through thiopurines and biologics soon after diagnosis.

Data relating to the proportion of patients with IBD being treated with thiopurines are lacking. The IBD BioResource recruits in over 100 hospitals covering the spectrum from small district hospitals to large university centres. In this cohort, broadly representative of UK hospital practice, it is clear that thiopurines have been and continue to be widely used—in 43.8% of patients with UC and 71.9% with CD. Interestingly the proportion on thiopurines does not appear to have reduced significantly with increasing biological therapy availability (data not shown). National Institute for Health and Care Excellence recommendations may be relevant to this, as in UK practice all patients escalated to biologics should have first trialled immunomodulators.

A key question with any drug in IBD is when and whether to stop treatment in those achieving remission. Previous small studies considered thiopurine cessation appropriate after 4 years but relapse following cessation is common.^13 26^ Strikingly, in the current study, most patients in whom treatment was deemed effective were still on thiopurines at the data lock, with a median treatment duration of 16 (CD) and 17 (UC) years on Kaplan-Meier analysis (online supplementary figure 4). It seems that thiopurines are continued for prolonged periods where they are proving effective despite well-documented concerns about increased risk of skin cancers and lymphoma long term.^34^

Early RCTs of thiopurines in IBD included only modest numbers of patients and short follow-up—and one or both of these limitations also apply to subsequent ‘real-world’ studies.^4–10 13–15 17 18^ A recent meta-analysis of 489 CD subjects showed remission rates of 73% on AZA, but also 62% in the placebo group (RR 1.19, 95% CI 1.05 to 1.34) with limited follow-up of 6–18 months.^1^ Jharap *et al* reported thiopurine effectiveness of 48% for CD and 38% for UC in two 8 years intercept cohorts totaling 366 patients.^15^ However, effectiveness was defined as clinical remission in patients still on thiopurine at 5 years, regardless of surgery or anti-TNF commencement. Another ‘real-world’ study from Oxford undertook retrospective case notes review on 622 thiopurine-experienced IBD patients.^13^ Relapse was defined as need for steroids or surgery, and the assessment of effectiveness required at least 6 months on azathioprine, therefore, patients unable to tolerate the medication were excluded from this analysis. Thiopurines appeared effective in 45% of patients with CD and 58% with UC but average treatment duration was less than 2 years.

RCTs of thiopurine in UC are even more limited. A meta-analysis of six studies included only 124 patients and suggested 60% remission on thiopurine vs 37% on placebo or 5 aminosalicylates.^35^ Observational studies have typically included 100–250 patients and reported benefit in 40%–60%.^13 15 36–38^

With 11 928 participants contributing 68 132 years of thiopurine exposure data, the large size of our study should underscore confidence in our findings. Nevertheless, our study clearly also has limitations. Necessarily for a large-scale retrospective study we used a pragmatic, but non-validated definition of effectiveness which may not always equal remission. This required a clinical judgement regarding effectiveness and also persistence on thiopurine without need for biological escalation or surgery. Studies frequently now use persistence on drug as a marker of continued effectiveness, so in this regard our definition was more rigorous than many.^39 40^

Other limitations include the lack of assessment of mucosal healing for example by calprotectin assay or endoscopy data. The IBD BioResource is a hospital-based programme, and hence may be skewed towards individuals with more severe disease. This will have inflated the proportion of patients with IBD who have been treated with thiopurines and indeed biologics compared with population cohorts. In addition, all patients on thiopurines, regardless of dose, were included. We have not captured data on thiopurine dosing or metabolite monitoring to identify patients in whom thiopurines were ineffective due to underdosing. Nor have full data on concomitant use of 5-ASA, or the need for corticosteroids or hospitalisation been captured. However, we feel it unlikely that 5-ASA use alone would explain the differences we observed; and in UK practice recurrent need for either corticosteroids or hospitalisation in patients established on thiopurine would conventionally mandate escalation to biologic therapy or surgery—data which we did capture to signal lack of thiopurine effectiveness.

In UC, we observed a colectomy rate of 4.4% in individuals in whom thiopurine monotherapy was tried (and 5.1% in all patients with UC in the IBD BioResource). Although lower than many historic series,^41–45^ two recent British cohorts have reported similar rates. Chhaya *et al* reported that in a population cohort of 8673 incident UC patients (1766 receiving thiopurines) 5.5% underwent colectomy,^20^ while Alexakis *et al* reported a 4.9% crude colectomy rate.^46^ In common with the former study, we sought evidence that thiopurines reduced UC colectomy risk. By comparing patients able to tolerate the medication with those who had been treated with thiopurine but were unable to tolerate it we controlled for the impact of disease severity on colectomy risk (selection bias). Our regression analysis showed a significantly earlier need for surgery in thiopurine-intolerant patients (figure 4), with an HR of 2.44 for risk of earlier colectomy (table 4). Consistent with other studies our data also showed higher colectomy risk in extensive versus distal UC. Chhaya *et al* identified a 71% reduction colectomy risk in 1766 individuals with UC treated with thiopurine for at least 12 months compared those on it for less than 12 months. Similar conclusions were drawn from a population-based study from Manitoba and a recent Scandinavian hospital cohort (reduction in absolute colectomy risk from 29% to 19.5%).^20 21 41^

Evidence in our study for the benefits of thiopurine therapy on the need for surgery in CD was more modest than in UC (figure 5 and table 5). We observed a lower risk of abdominal surgery in older patients and in those with colonic disease without small bowel involvement, as previously reported.^13^ In two recent studies, one a Danish CD population cohort of 13 185 patients, thiopurine use did not appear to affect surgical rates^46 47^; but others suggest that thiopurines do reduce surgery in CD—for example, a UK population cohort study by Chatu *et al* and data from Hungary.^25 48^ Our data revealed the same direction of effect as the earlier UK study but more modest benefit in reducing surgery. Substantial differences in study design may account for this difference, such as patient selection (population vs hospital-based cohorts), capturing of surgery (unlike Chatu *et al* we included all IBD-related surgery including perianal CD surgery) and eras examined (we included patients diagnosed pre-1989 and post-2005). In a 2014 meta-analysis, thiopurine use correlated with a 40% reduced risk of resectional surgery in CD.^49^ However, asymmetry was observed in the funnel plot, with studies showing no benefit of thiopurines in surgery being under-represented. Additionally, this meta-analysis did not include the large, negative Danish study published shortly after.^47^

## Conclusion

The current study provides robust real-world evidence from the large UK IBD BioResource dataset that thiopurine monotherapy is an effective long-term treatment for UC. However, it appears less effective in CD (OR=0.47) particularly where there is perianal involvement. Previous studies showed no benefit of thiopurines initiated very soon after CD diagnosis (with consistent data in our study),^11 12^ and there is clear evidence from trials such as SONIC for improved efficacy when thiopurines are combined with infliximab for the treatment of CD.^50^ Given recent substantial reductions in the cost of biosimilar anti-TNF therapy, and the evidence presented regarding the relative lack of durable effectiveness of thiopurine monotherapy in CD, there is perhaps a need to re-evaluate treatment strategies. For patients with anything more than mild to moderate CD thiopurines should perhaps be viewed primarily as an adjunct to anti-TNF therapy.
