# Supplementary material for: Thiopurine monotherapy is effective in ulcerative colitis but significantly less so in Crohn’s disease: long-term outcomes for 11 928 patients in the UK inflammatory bowel disease bioresource
Source: Gut. 2020 Oct 1;70(4):677–86. doi: 10.1136/gutjnl-2019-320185 (PMC7948184; doi:10.1136/gutjnl-2019-320185)
Supplement: Supplementary data [file gutjnl-2019-320185supp003.pdf]

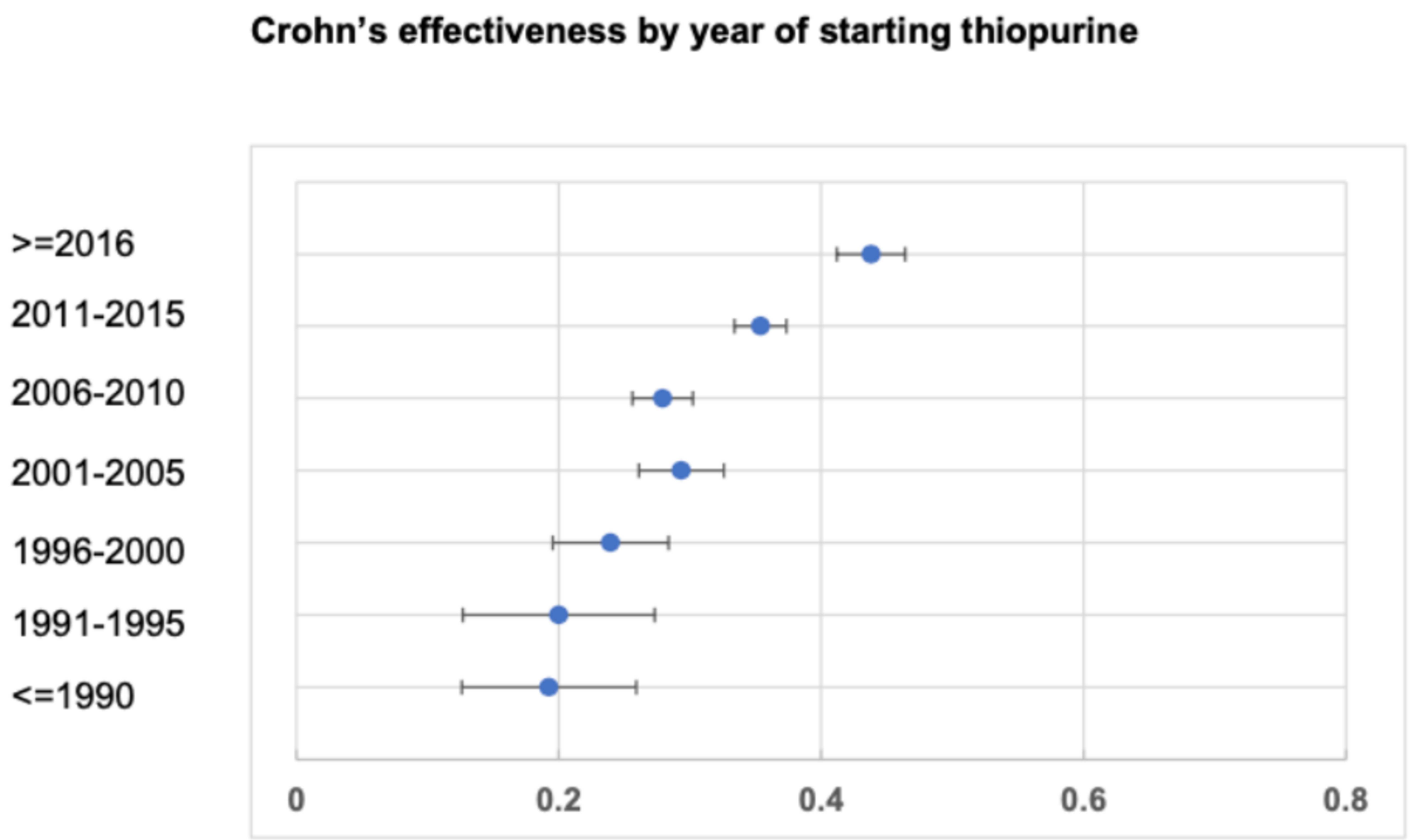

Supplementary Figure 3. Proportion of CD patients in whom thiopurine monotherapy was deemed effective, stratified by period of starting treatment
