# Supplementary material for: Thiopurine monotherapy is effective in ulcerative colitis but significantly less so in Crohn’s disease: long-term outcomes for 11 928 patients in the UK inflammatory bowel disease bioresource
Source: Gut. 2020 Oct 1;70(4):677–86. doi: 10.1136/gutjnl-2019-320185 (PMC7948184; doi:10.1136/gutjnl-2019-320185)
Supplement: Supplementary data [file gutjnl-2019-320185supp004.pdf]

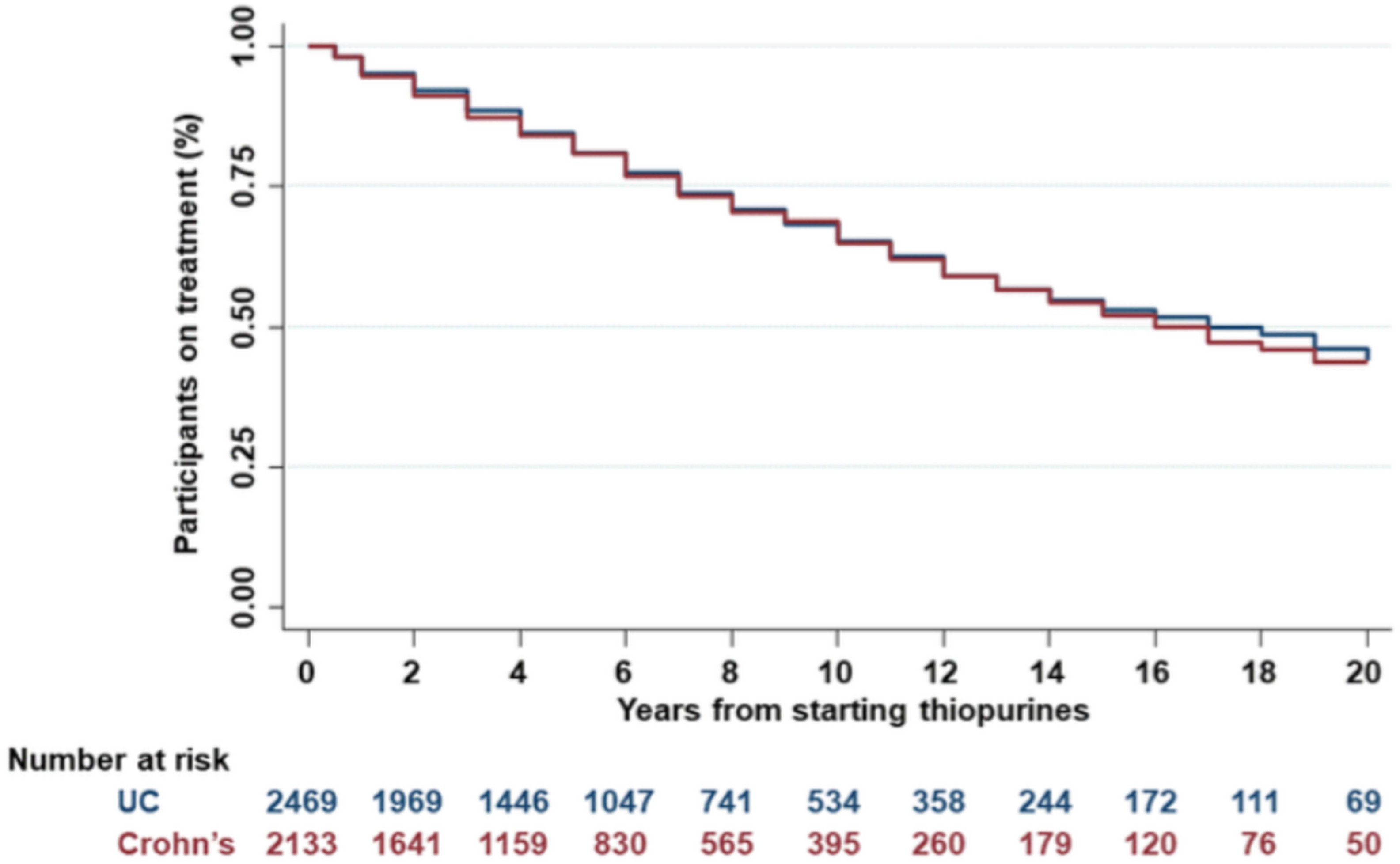

Supplementary Figure 4. Kaplan-Meier plot showing duration of thiopurine treatment in patients in whom thiopurine monotherapy was deemed effective. Median is 17 years (95%CI=15-20) for UC and 16 years (95% CI=16-20) for CD.
