# Supplementary material for: Thiopurine monotherapy is effective in ulcerative colitis but significantly less so in Crohn’s disease: long-term outcomes for 11 928 patients in the UK inflammatory bowel disease bioresource
Source: Gut. 2020 Oct 1;70(4):677–86. doi: 10.1136/gutjnl-2019-320185 (PMC7948184; doi:10.1136/gutjnl-2019-320185)
Supplement: Supplementary data [file gutjnl-2019-320185supp005.pdf]

Supplementary table 1. Percentage (%) of patients not requiring surgery/biologics at 1,2,3,5,10,15,20 years after diagnosis.

		Crohn's	UC
All patients		N=6464	N=4725
Median time (years) without treatment escalation (biologics or surgery) (95%CI)		4 (4, 5)	12 (11, 13)
P25, P75		1, 12	2, 30
Percentage of patients without treatment escalation after N years of thiopurine monotherapy initiation	N=1	69%	76%
	N=2	60%	72%
	N=3	54%	69%
	N=5	45%	64%
	N=10	29%	53%
	N=15	19%	43%
	N=20	13%	37%
